# Bio-efficacy of DuraNet® long-lasting insecticidal nets against wild populations of *Anopheles arabiensis* in experimental huts

**DOI:** 10.1186/s41182-018-0118-5

**Published:** 2018-11-06

**Authors:** Aneth M. Mahande, Shandala Msangi, Lucile J. Lyaruu, Eliningaya J. Kweka

**Affiliations:** 1 Division of Livestock and Human Diseases Vector Control, Tropical Pesticides Research Institute, Mabogini Field Station, Moshi, Tanzania; 2 Division of Livestock and Human Diseases Vector Control, Mosquito Section, Tropical Pesticides Research Institute, P.O. Box 3024, Arusha, Tanzania; 3 Department of Medical Parasitology and Entomology, School of Medicine, Catholic University of Health and Allied Sciences, P.O. Box 1464, Mwanza, Tanzania

**Keywords:** Behavior, Long-lasting insecticidal nets, Mosquito, Northern Tanzania

## Abstract

**Background:**

Mosquitoes have developed resistance to multiple classes of insecticides for malaria vector control. A new generation of long-lasting insecticidal bed nets (LLINs) has been developed with increased efficacy against these resistant mosquitoes. The present study therefore evaluated the efficacy of the pyrethroid-based LLINs, DuraNet versus PermaNet 3.0, in an Eastern Africa hut design in Magugu in northern Tanzania where mosquitoes’ population higher proportion (69.3%) has been identified as *Anopheles gambiae* s.l.

**Methods:**

Standard World Health Organization bioefficacy evaluations were conducted in both laboratory and experimental huts. Experimental hut evaluations were conducted in an area with high populations of *Anopheles arabiensis*. All nets used were subjected to laboratory cone bioassays and then to experimental hut trials. Mosquito mortality, blood-feeding inhibition, and personal protection rate were compared between untreated nets, unwashed LN, and LN that were washed 20 times.

**Results:**

Standard WHO laboratory bioefficacy evaluations of DuraNet and PermaNet® 3.0 which were untreated, washed, or 20 times washed showed optimal knockdown and mortality for both net types against a susceptible strain of *An. arabiensis*. In standard experimental hut evaluations, the blood feeding inhibition for PermaNet® 3.0 unwashed and washed was 82.4% (76.3–88.6%) to 91.5% (84.1–98.8%) while for DuraNet was 98.3% (97.0–99.5%) to 96.0% (94.1–88.2%) respectively. The DuraNet LLINs showed a significantly higher killing effect than the other treatment of 90.0% (86.1–94.2%) and 94.0% (90.2–97.9%) for unwashed and washed nets respectively. No significant difference in deterrence or induced exophily was detected between the treatment arms. There were no adverse effects reported among sleepers in the experimental huts.

**Conclusion:**

The findings of this study indicate that the pyrethroid-based net DuraNet LLINs attained required efficacy when evaluated against wild population of *An. arabiensis* from Northern Tanzania. This adds value to the existing vector control tool box which gives community wider choice for vector control.

## Introduction

Malaria control efforts with long-lasting insecticidal nets (LLINs) and conventionally treated nets took a new phase of implementation since 2000 with a great impact in decline of disease incidences and mortality [1, 2]. Mortality cases have declined to 438,000 cases per year in 2015 from formally 2.7 million in 2000 worldwide [1, 2]. Also, this decline in malaria has been attributed by other several malaria control measures used such as artemisinin combined therapy (ACT), Intermittent Preventive Treatment (IPTP) for pregnant women and vector control including long-lasting insecticides nets (LLINs), and indoor residual spraying of insecticide (IRS) [1, 2].

A long-lasting insecticidal net (LLIN) is “a factory-treated mosquito net that is expected to retain its biological activity after a minimum number of standard washes and a minimum period of use under field conditions” [3]. LLIN remains the key for malaria vector control in rural and urban settings in Tanzania and is more effective in malaria prevention when there is a full coverage of all people at risk [4–8]. In addition, LLINs have been reported to be more cost-effective than IRS programs with the average IRS cost/person/year protection of $ 2.62 as compared to $ 1.39 for LN in 3 years’ time [9, 10]. LLINs have been found to reduce malaria morbidity and mortality at the community level in malaria-endemic countries worldwide [11–14]. But despite the wide use of LLINs in Tanzania, where over 18 million LLINs have been distributed by the Ministry of Health and partners since 2004, malaria remains the major health problem in Tanzania [15, 16]. The wide coverage of LLINs is thought to have contributed to the development of insecticide resistance in the local malaria vectors population due to the insecticide selection pressure encountered by vectors [9, 17]. Many vector species have developed resistance to one or more classes of insecticides commonly used in Tanzania [18–20]. Development of vector resistance may be caused by many factors including overuse of insecticides by ITNs, IRS, and household pest control or by agricultural pesticides use [19, 21–24]. The WHO global insecticide resistance database has reported pyrethroid resistance detection in 78% of the countries that have reported monitoring data, and 80% of these countries have reported resistance in two or more classes of insecticides [25].

The effect of insecticide resistance has been reported to reduce the LLIN effectiveness and increase malaria risks in areas with high insecticide resistance for mixed classes of insecticide [26]. In Northwest of Tanzania, insecticide resistance of Bendiocarb (Carbamates) against *Anopheles gambiae* s.l. was reported and found a sharp reduction in mosquito mortality from 84% in December 2011 to 31% in December 2012 [18, 27]. Similarly, pyrethroids resistance against *An. arabiensis* and *An. gambiae* s.s. has been reported in different parts of Tanzania, such as Muleba, Mabogini, Arumeru, and Muheza [18, 26, 27]. Efforts are underway to develop new vector control products with different modes of action against mosquito that will effectively control resistant strains to currently used classes of insecticide.

In this study, the pyrethroid (alphacypermethrin, 5.8 g of active ingredients/kg ± 25%)-based LLINs DuraNet® was evaluated in experimental huts in comparison to pyrethroid-synergist PermaNet 3.0 and an untreated net. The work was done following the standard WHO procedures [28] to determine comparative efficacy against a free-flying wild resistant population of *An. arabiensis* as per standard measured outcomes from experimental hut trials. The LLIN efficacy was measured in terms of blood-feeding inhibition, deterrence, induced exophily, and mortality.

## Materials and methods

### Study sites

This study was conducted during July–December 2015, in malaria epidemic-prone site, Magugu ward, Babati district in Manyara region [29]. The area is located in the Great Rift Valley of northern Tanzania (3° 53′ S, 35° 42′ E) which is 150 km from Arusha Town. The main economic activities include livestock keeping and crop cultivation mainly maize and rice farms. This area has seasonal malaria prevalence and sometimes epidemics caused mainly by *Plasmodium falciparum* transmitted by *Anopheles gambiae* s.l. mosquitoes during the rainy season and transmitted by *Anopheles funestus* mosquitoes during the dry season [30]. Malaria vector susceptibility status to pyrethroid insecticides was found to have mortality ranging from 98 to 99%, during susceptibility tests conducted in 2011 [31].

### Hut design

The experimental huts used were of East Africa design, which were constructed with burnt bricks and roved with corrugated iron sheets. These included a double verandah trap resembling that described by Smith and others [32] with brick walls plastered with mud on the inside, a wooden ceiling lined with hessian sackcloth, open eaves, and window traps and verandah traps on each side. The huts were built on concrete plinths and surrounded by a water-filled moat to deter entry of scavenging ants. Mosquitoes fly upward to enter through the eaves and then fly downwards to exit; this precludes or greatly limits exodus through the aperture enabling the majority of entering mosquitoes to be accounted for. Two opposite sides of the huts had closed verandas, screened to capture mosquitoes that left via the eaves. The other two verandas were left open so mosquitoes could enter through the eaves.

Each night’s collection inside the two screened verandah traps was multiplied by two and added to the room and window trap collections. The multiplication is to adjust for the unrecorded escapes through the two verandas which were left unscreened to allow routes for entry of wild mosquitoes via the 4-cm gaps under the eaves [32].

### Treatment arms

Washed and unwashed LLINs were evaluated using experimental huts for their effects on free-flying wild mosquitoes and for their ability to deter entry, repel or drive mosquitoes out of houses, induce mortality, and inhibit blood-feeding. PermaNet 3.0 was used as a positive control, and untreated polyester net was used as a negative control. The following treatment arms were tested using six nets per arm for the study:

1. Untreated polystyrene net
2. PermaNet® 3.0 unwashed
3. PermaNet® 3.0 washed 20 times
4. DuraNet® unwashed
5. DuraNet® washed 20 times

Before testing in the experimental huts, the nets (including the untreated control) were deliberately holed. Six holes were made in each net, two holes in each of the long side, and one hole at each end. Each hole measured 4 cm × 4 cm. Each net was individually coded with two numbers: treatment number and net number.

### Net washing procedure

The nets were washed according to a protocol adapted from the standard WHO washing procedure used in phase II, over a 30-day period interval (i.e., by applying the regeneration time value that was established under phase I of 24 h) [33]. Nets were washed in aluminum bowls containing 10 l of well water having a maximum hardness of 5 dh and containing 2 g/l of soap (“Savon de Marseille”) using manual agitation. For each wash, nets were agitated for 3 min, left to soak for 4 min, and re-agitated for 3 min for a total of 6-min agitation during a 10-min washing/soaking period. Agitation was done by stirring the net with a pole at 20 rotations per minute. Rinsing was done twice using clean water (10 l per rinsing, i.e., 20 per net). Nets were dried horizontally in the shade then stored at ambient temperature between washes.

### Cone bioassays

Six nets (one net per treatment arm, including the untreated control) of each treatment were bio-assayed the day before the first wash. Bioassays were done again for a second time when all washings were completed and for a third time at the end of the field experiment with nets used in huts. Bioassays were conducted according to the WHO procedures for cone tests [28]. For each net, five cones were placed on the five sections of the net (roof and four sides). Five (5) non-blood fed females of *An. gambiae* susceptible Kisumu strain from TPRI insectaries were introduced per cone and exposed for 3 min to the net surfaces. Bioassays were replicated five times for a total of 25 mosquitoes tested per position on each net. Knockdown was recorded 60 min after exposure, and mortality was scored 24 h after exposure. During the observation period, mosquitoes were provided with 10% sugar solution.

### Experimental hut study design

One net was hung in each hut, with one net per treatment type deployed concurrently in the five test huts. Each week, the treatment arms were rotated among the huts according to a Latin square scheme. Six nets were used per treatment arm, and each of the six nets was tested one night during the week. At the end of the week, the huts were carefully cleaned and aired for 1 day to remove potential contamination. The treatment was then rotated to a different hut. The trial continued for 6 weeks to ensure complete rotation through the huts. In this study, 6 weeks, i.e., one complete Latin square, was long enough to obtain sufficient numbers of mosquitoes for adequate statistical analysis.

In each of the hut, one sleeper occupied a bed and slept inside the tested net during the night as from 18:00 to 06:00 in the morning. Each morning, between 06:00 and 08:00 am, two experienced mosquito technicians collected all dead mosquitoes on floors, those resting inside, verandah traps, and window traps of each hut using hand aspirators for estimating deterrence and exophily. The collected mosquitoes were put separately in paper cups, sorted into their live status and abdominal conditions and then identified to species level using identification key [34, 35]. The killing effect was observed for all mosquitoes collected from each treatment arms.

### Perceived side effects

The sleepers in the huts were interviewed at the end of the experiment about perceived adverse or beneficial side effects of the LLINs and of the untreated nets when implemented in accordance with standard procedures [28]. The hut sleepers were recruited from the study area and were requested to notice any noticeable adverse side effects in the course of the whole study. A short questionnaire was prepared and used to seek information from the hut sleepers regarding possible itching, dizziness, or running nose. Hut sleepers were given questionnaire to respond every morning slept in each net brand. Each hut had one adult sleeper as recommended in huts trial for bed nets in WHO protocol, and they were rotated within huts to avoid the biasness effect on mosquito attraction [33].

### Statistical analysis

The proportion of mosquitoes that exited early, the proportion that were killed within the hut, and the proportion that successfully blood fed were compared by species and then analyzed by logistic regression or generalized linear mixed models (using Statistical Package for Social Scientists (SPSS version 25(SPSS Inc., Chicago, IL)), which provide a framework for regression modeling of non-normal outcome data (such as mosquito mortality) while naturally adjusting for clustering effects. The clustering of observations made in one hut night and any variation between huts and sleepers was controlled for by adjusting the models.

Outcome measured was calculated as per standard procedures [28]. The primary analysis was a test of the non-inferiority of the DuraNet washed 20 times relative to PermaNet® 3.0 unwashed and that washed 20 times. According to WHOPES, a candidate LLINs is considered to meet the phase II efficacy criteria if, after 20 washes, it performs as well as or better than the reference LLINs when washed 20 times in terms of blood feeding inhibition and mortality.

The mean numbers of unfed and fed mosquitoes from indoor, verandah, and window traps from treated and untreated huts were compared, and the induced exophily was calculated using the Abbott formula [36] [(*N*_c_ − *N*_t_)/*N*_c_] × 100%, where *N*_t_ is the number of mosquitoes from verandah and window traps of treated hut while *N*_c_ is the number of mosquitoes from verandah and window traps of untreated hut. Similarly, feeding inhibition was estimated using the formula [(*F*_c_ − *F*_t_)/*F*_c_] × 100%, where *F*_c_ is the number of mosquitoes found fed in untreated control hut while *F*_t_ is a number of mosquitoes found fed in treated hut.

## Results

### Species composition

A total of 1020 mosquitoes were collected in five experimental huts. Of all the mosquitoes collected, *An. arabiensis* mosquitoes were the most predominant (69.3%) species. Other mosquito species collected were *Culex quinquefasciatus* and *Mansonia spp.* which made 29.6% of the population.

### Bioefficacy of nets in laboratory

**Fig. 1:**
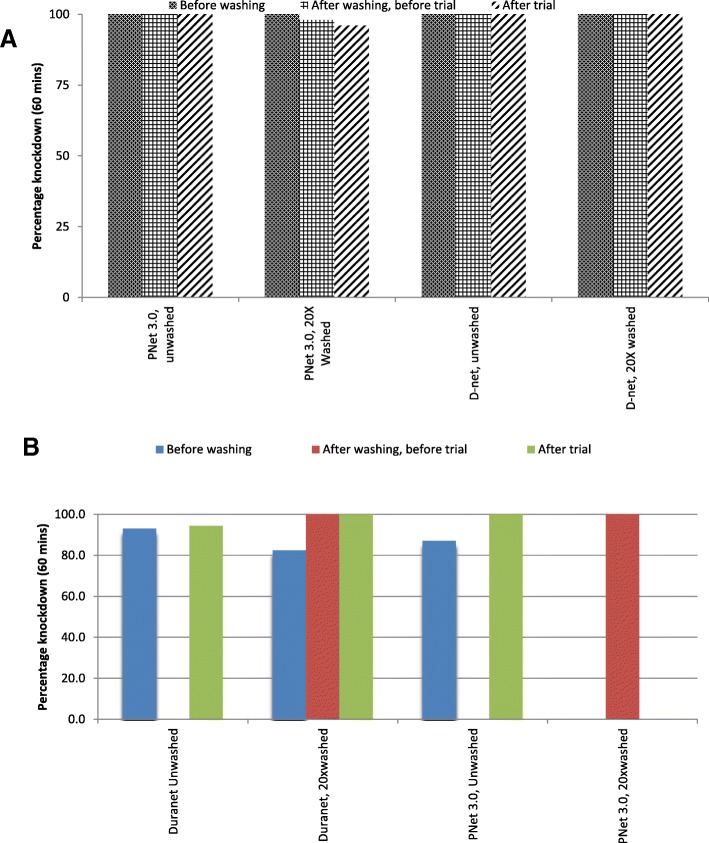
Bioefficacy (percentage knockdown) in WHO cone bioassays of sub-samples from LN against **a** a susceptible laboratory strain and **b** resistant field strain of *An. arabiensis*

**Fig. 2:**
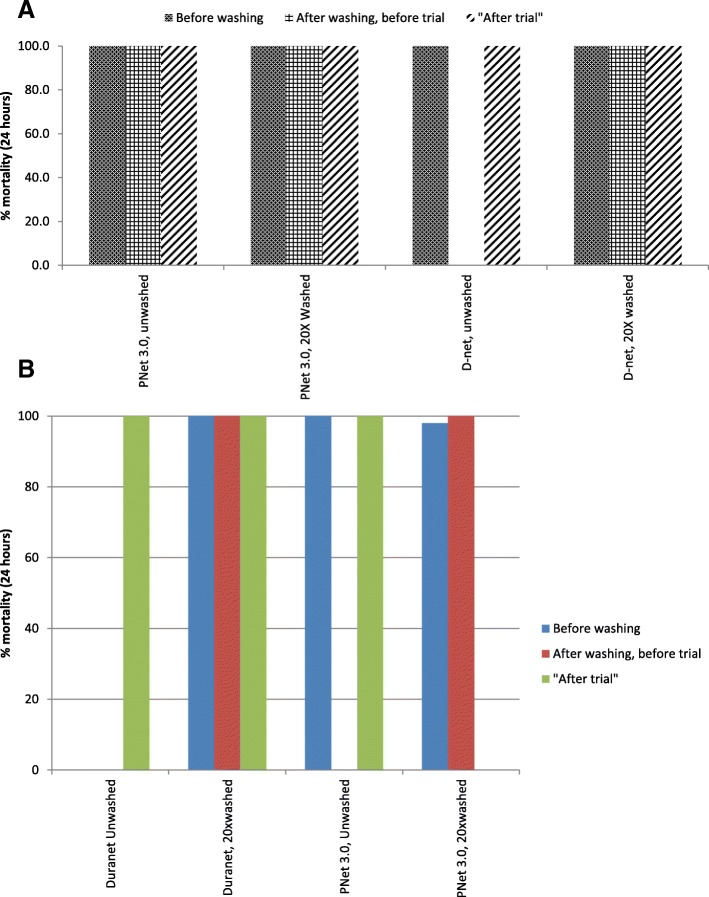
Bioefficacy (percentage mortality) in WHO cone bioassays of sub-samples from LN against **a** a susceptible laboratory strain and **b** a resistant field strain of *An. arabiensis*

Efficacy testing of LLIN sub-samples via cone bioassays with a susceptible laboratory strain of *An. gambiae s.s.* (Kisumu) indicated that DuraNet and PermaNet® 3.0 had optimal bioefficacy at all three time points, including before washing, after washing and before the trial, and after the trial (Fig. 1a, b and 2a, b). All nets therefore surpassed WHO bioefficacy cutoffs of ≥ 80% mortality or ≥ 95% knockdown.

### Bioefficacy of nets in experimental hut trial

In the experimental huts trial, there was no significant difference in the number of mosquitoes entering each of the treatment arms therefore indicating that there was no difference in deterrence among treatments except for unwashed DuraNet LN. Induced exophily ranged from 86.9% (83.6–90.1%) to 89.1% (87.2–91.9%) for both DuraNet® washed and unwashed. However, there was an apparent significant difference in induced exophily when compared PermaNet 3.0 and DuraNet either washed or unwashed (Table 1).

**Table 1:** The experimental huts response for *Anopheles arabiensis* collected during the 6 weeks trial for the behavioral analysis of DuraNet and PermaNet3.0 unwashed and washed

Parameter	Summary data	Untreated net (u)	DuraNet, unwashed	DuraNet, washed 20 times	PermaNet 3.0, unwashed	PermaNet 3.0, washed 20 times
Deterrence	Total number of females caught	385	84	257	241	169
	Females caught/night	15.4	3.4	10.3	9.6	6.8
	Deterrence (%)	0	78.18	33.25	37.40	56.10
Exophily	Number females in exit traps and verandah	118	73	229	188	131
	Exophily (%)	30.6	86.9	89.1	78.0	77.5
	95% confidence limits	26.6–35.3^a^	83.6–90.1^b^	87.2–91.9^b^	76.3–80.2^c^	74.2–81.1^c^
Blood feeding	Number of blood-fed females (B)	176	3	7	31	15
	Percentage of blood-fed	45.7	3.6	2.7	12.9	8.9
	95% confidence limits	41.1–51.1^a^	1.9–4.9^b^	1.3–4.0^b^	10.3–15.3^c^	5.9–12.3^c^
	Blood feeding inhibition (%)	0	98.3	96.0	82.4	91.5
Mortality	Number dead females morning (immediate mortality)	0	4	8	6	5
	Number dead females after 24 h (delayed mortality)	1	6	3	4	5
	Total number dead females (K)	1	10	11	10	10
	Overall mortality (%)	0.3	11.9	4.3	4.1	5.9
	95% confidence limits	0.11–0.43^a^	8.8–14.8^b^	2.5–6.0^c^	2.4–5.1^c^	2.8–8.7^c^
	Mortality corrected for control (%)		11.7	4.0	3.9	5.7
Summary	Personal protection (%)		98.3	96.0	82.4	91.5
	Killing effect (%)		90.0	100	90.0	90.0

Those 95% CI values in the same raw with different superscript letters differ significantly

Blood feeding was 45.7% (41.1–51.1%) higher for the untreated control than for the treatment arms, and the calculated personal protective effect was 82.4% (76.3–88.6%) to 91.5% (84.1–98.8%) for both PermaNet® 3.0 unwashed and washed while for DuraNet ranged between 96.0% (94.1–88.2%) and 98.3% (97.0–99.5%) for 20 times washed and unwashed nets respectively (Table 1). Mortality corrected for controls was highest for DuraNet unwashed and least in PermaNet 3.0 unwashed (4.3% (2.4–6.1%) to 11.9% (8.8–15.3%)). The overall killing effect of nets varied between unwashed and washed 20 times DuraNet and PermaNet3.0 and was significantly (*P* = 0.032) highest for DuraNet washed 20 times (Table 1).

### Perceived side effects

There were no reported negative side effects such as itching, dizziness, or nose running among the five hut sleepers who participated in the interview for the all experimental days.

## Discussion

The current study has found the wild population been composed of *An. arabiensis* by 69.3% and 29.6% was made up by *Cx. quinquefasciatus* and *Mansonia spp*. This species composition is similar to what has been found by previous studies conducted in Magugu formerly Umbugwe land [30, 37, 38].

The findings of this study have shown that the lowest deterrence was observed in DuraNet washed 20 times and PermaNet 3.0 unwashed while the highest was found in DuraNet unwashed (78%). The low deterrence rate observed in this current study was similar to what was observed in other study based on PermaNet 3.0 nets evaluation [19, 39]. The findings of this study have revealed that the deterrence of DuraNet evaluated is comparable to standard nets of PermaNet 3.0 when tested against wild free-flying mosquitoes [19, 39]. In the study conducted in India, DuraNet had deterrence similar to other LLINs such as Olyset, Netprotect, PermaNet, and interceptor [40]. This study results have revealed that the DuraNet can be widely use as physicals and chemical barriers against malaria vectors. This is highly concurring with study conducted in Solomon Island which has shown that community acceptance of DuraNet usage is 68.7% [41]; hence, community protection with deterrence efficacy shown can have impact on malaria decline.

The blood feeding inhibition ranged between 82.4 to 98.3% for treated nets compared to control. In previous studies, it was observed that unwashed PermaNet 3.0 nets had feeding inhibition efficiency of 91% while after 20 times washes had 63% feeding inhibition [39]. The evaluated nets (DuraNet) had blood feeding inhibition efficiency of 98.3% before being washed and dropped to 96% after 20 times washes but was not as drastic as that of PermaNet 3.0 found in Muheza [39]. The study conducted in India has shown DuraNet to have similar feeding inhibition as other long-lasting nets such as Olyset, Netprotect, PermaNet, and interceptor [40]. The increased blood feeding inhibition has shown to have potential of adding value to the currently existing tools against malaria vectors.

In the assessment of the knockdown effect in laboratory before wash, after 20 times washes, and after experimental hut trial, the knockdown shown was above accepted WHO cutoff point (above 80%) [42]. There was reduced knockdown in all stages of cone bioassays using wild resistant population of *An.arabiensis*. This trend seemed similar to previous study conducted in areas with resistant wild population with reported resistant population in pyrethroids, organophosphates, and carbamates [31]. In this study, the mortality effect of the mosquitoes collected in experimental huts with unwashed net was 11.7% while with 20 times washed net was 3.9%. This was contrary to what was found in trial conducted in India which had mortality effect of 74.5% [40]. The lower mortality effect might have been attributed with increased insecticides resistance in Tanzania vector population for both washed and unwashed DuraNet [18, 26, 27, 31]. This is also similar to numerous experimental hut studies evaluated different net brands in Tanzania [9, 18, 26, 27, 39], and hence, the most important outcomes from such trials were the mortality parameters which are summarized by personal protection and killing effect outcomes [19, 43, 44]. The overall killing effect was found to be significantly higher for DuraNet washed 20 times than unwashed; this might be attributed with the high susceptibility status to variety of the insecticides in this study area [31].

The current findings have shown to have exophily rate of 86.9% when unwashed and 89.1% after 20 times wash. The recorded exit rate in this study was found to be higher than that recorded in previous in India [40]. This might be attributed with the variance in species composition between the study sites involved and variations in insecticides tolerance.

During the trial, there was no any adverse effect reported among experimental hut sleepers and mosquito sampling individuals in the huts where DuraNet was used. Similar response was found with the study conducted in Asia with DuraNet acceptability rate found to be 69.8% [41].

## Conclusion

Based on these findings, DuraNet has shown protection efficiency against wild population with mortality high effect in huts. This has added an additional tool in malaria vector control toolbox and selection option.

## Abbreviations

ACT: Artemisinin combined therapy
IPTP: Intermittent preventive treatment
IRS: Indoor residual spray
LLINs: Long-lasting insecticidal nets

## References

[1] WHO (2016). World malaria report 2016.

[2] WHO (2017). World malaria report 2017.

[3] WHO, "Guidelines for laboratory and field testing of long-lasting insecticidal mosquito nets. WHO/CDS/WHOPES/GCDPP/2005.11," 2005.

[4] Njau JD, Stephenson R, Menon M, Kachur SP, McFarland DA (2013). Exploring the impact of targeted distribution of free bed nets on households bed net ownership, socio-economic disparities and childhood malaria infection rates: analysis of national malaria survey data from three sub-Saharan Africa countries. Malar J.

[5] Wanzira H, Yeka A, Kigozi R, Rubahika D, Nasr S, Sserwanga A (2014). Long-lasting insecticide-treated bed net ownership and use among children under five years of age following a targeted distribution in central Uganda. Malar J.

[6] Protopopoff N, Wright A, West PA, Tigererwa R, Mosha FW, Kisinza W (2015). Combination of insecticide treated nets and indoor residual spraying in northern Tanzania provides additional reduction in vector population density and malaria transmission rates compared to insecticide treated nets alone: a randomised control trial. PLoS One.

[7] Ossè RA, Aïkpon R, Gbédjissi GL, Gnanguenon V, Sèzonlin M, Govoétchan R (2013). A shift from indoor residual spraying (IRS) with bendiocarb to long-lasting insecticidal (mosquito) nets (LLINs) associated with changes in malaria transmission indicators in pyrethroid resistance areas in Benin. Parasit Vectors.

[8] Sovi A, Azondékon R, Aïkpon RY, Govoétchan R, Tokponnon F, Agossa F (2013). Impact of operational effectiveness of long-lasting insecticidal nets (LLINs) on malaria transmission in pyrethroid-resistant areas. Parasit Vectors.

[9] Abílio AP, Marrune P, de Deus N, Mbofana F, Muianga P, Kampango A (2015). Bio-efficacy of new long-lasting insecticide-treated bed nets against Anopheles funestus and Anopheles gambiae from central and northern Mozambique. Malar J.

[10] Lee BY, Bartsch SM, Stone NT, Zhang S, Brown ST, Chatterjee C (2017). The economic value of long-lasting insecticidal nets and indoor residual spraying implementation in Mozambique. Am J Trop Med Hyg.

[11] Flaxman AD, Fullman N, Otten MW, Menon M, Cibulskis RE, Ng M (2010). Rapid scaling up of insecticide-treated bed net coverage in Africa and its relationship with development assistance for health: a systematic synthesis of supply, distribution, and household survey data. PLoS Med.

[12] Banek K, Lalani M, Staedke SG, Chandramohan D (2014). Adherence to artemisinin-based combination therapy for the treatment of malaria: a systematic review of the evidence. Malar J.

[13] Banek K, Kilian A, Allan R (2010). Evaluation of Interceptor long-lasting insecticidal nets in eight communities in Liberia. Malar J.

[14] Banek K, Nankabirwa J, Maiteki-Sebuguzi C, DiLiberto D, Taaka L, Chandler CI (2015). Community case management of malaria: exploring support, capacity and motivation of community medicine distributors in Uganda. Health Policy Plan.

[15] Mangham LJ, Hanson K (2010). Scaling up in international health: what are the key issues?. Health Policy Plan.

[16] Masum H, Shah R, Schroeder K, Daar AS, Singer PA (2010). Africa’s largest long-lasting insecticide-treated net producer: lessons from A to Z Textiles. BMC Int Health Hum Rights.

[17] Norris LC, Main BJ, Lee Y, Collier TC, Fofana A, Cornel AJ (2015). Adaptive introgression in an African malaria mosquito coincident with the increased usage of insecticide-treated bed nets. Proc Natl Acad Sci.

[18] Matowo J, Kitau J, Kaaya R, Kavishe R, Wright A, Kisinza W (2015). Trends in the selection of insecticide resistance in Anopheles gambiae s.l. mosquitoes in northwest Tanzania during a community randomized trial of longlasting insecticidal nets and indoor residual spraying. Med Vet Entomol.

[19] Kweka EJ, Lyaruu LJ, Mahande AM (2017). Efficacy of PermaNet® 3.0 and PermaNet® 2.0 nets against laboratory-reared and wild Anopheles gambiae sensu lato populations in northern Tanzania. Infect Dis Poverty.

[20] Philbert A, Lyantagaye SL, Pradel G, Ngwa CJ, Nkwengulila G (2017). Pyrethroids and DDT tolerance of Anopheles gambiae s.l. from Sengerema District, an area of intensive pesticide usage in north-western Tanzania. Tropical Med Int Health.

[21] Nkya TE, Akhouayri I, Poupardin R, Batengana B, Mosha F, Magesa S (2014). Insecticide resistance mechanisms associated with different environments in the malaria vector Anopheles gambiae: a case study in Tanzania. Malar J.

[22] Nkya TE, Poupardin R, Laporte F, Akhouayri I, Mosha F, Magesa S (2014). Impact of agriculture on the selection of insecticide resistance in the malaria vector Anopheles gambiae: a multigenerational study in controlled conditions. Parasit Vectors.

[23] Kweka EJ, Himeidan YE, Mahande AM, Mwang'onde BJ, Msangi S, Mahande MJ (2011). Durability associated efficacy of long-lasting insecticidal nets after five years of household use. Parasit Vectors.

[24] Wanjala CL, Kweka EJ (2018). Malaria vectors insecticides resistance in different agroecosystems in Western Kenya. Front Public Health.

[25] Ranson H, N’Guessan R, Lines J, Moiroux N, Nkuni Z, Corbel V (2011). Pyrethroid resistance in African anopheline mosquitoes: what are the implications for malaria control?. Trends Parasitol.

[26] Kabula B, Tungu P, Malima R, Rowland M, Minja J, Wililo R (2014). Distribution and spread of pyrethroid and DDT resistance among the Anopheles gambiae complex in Tanzania. Med Vet Entomol.

[27] Protopopoff N, Matowo J, Malima R, Kavishe R, Kaaya R, Wright A (2013). High level of resistance in the mosquito Anopheles gambiae to pyrethroid insecticides and reduced susceptibility to bendiocarb in north-western Tanzania. Malar J.

[28] WHO. Guidelines for laboratory and field-testing of long-lasting insecticidal nets. WHO/HTM/NTD/WHOPES/2013.3. Geneva: WHO; 2013.

[29] Mboera L, Kitua A (2001). Malaria epidemics in Tanzania: an overview. Afr J Health Sci.

[30] Mwanziva CE, Kitau J, Tungu PK, Mweya CN, Mkali H, Ndege CM (2011). Transmission intensity and malaria vector population structure in Magugu, Babati District in northern Tanzania. Tanzan J Health Res.

[31] Kabula B, Tungu P, Matowo J, Kitau J, Mweya C, Emidi B (2012). Susceptibility status of malaria vectors to insecticides commonly used for malaria control in Tanzania. Tropical Med Int Health.

[32] Smith A (1965). A verandah-trap hut for studying the house-frequenting habits of mosquitos and for assessing insecticides. 2. The effect of dichlorvos (DDVP) on egress and mortality of Anopheles gambiae Giles and Mansonia uniformis (Theo.) entering naturally. Bull Entomol Res.

[33] WHO (2013). Guidelines for laboratory and field-testing of long-lasting insecticidal nets.

[34] Gillies TM, de Meillon DB. The Anopheles of Africa South of Sahara (Ethiopian Zoogeographic Region). Johannesburg, Republic of South Africa. Publ S Afr Inst Med Res. 1968:54;2.

[35] Gilles M, Coetzee M. A supplement to the Anophelinae of Africa south of the Sahara. Publ S Afr Inst Med Res. Johannesburg. 1987:55.

[36] Abbott WS. A method of computing the effectiveness of an insecticide. J Am Mosq Control Assoc. 1925;3:302–3. 3333059

[37] Smith A (1962). Studies on domestic habits of A. gambiae that affect its vulnerability to insecticides. East Afr Med J.

[38] Smith A (1962). The preferential indoor resting habits of Anopheles gambiae in the Umbugwe area of Tanganyika. East Afr Med J.

[39] Tungu P, Magesa S, Maxwell C, Malima R, Masue D, Sudi W (2010). Evaluation of PermaNet 3.0 a deltamethrin-PBO combination net against Anopheles gambiae and pyrethroid resistant Culex quinquefasciatus mosquitoes: an experimental hut trial in Tanzania. Malar J.

[40] Gunasekaran K, Sahu S, Vijayakumar T, Vaidyanathan K, Yadav R, Pigeon O (2014). Comparison of efficacy of five types of long-lasting insecticidal nets against Anopheles fluviatilis, the primary malaria vector in East-Central India. J Med Entomol.

[41] Atkinson J-A, Bobogare A, Vallely A, Boaz L, Kelly G, Basifiri W (2009). A cluster randomized controlled cross-over bed net acceptability and preference trial in Solomon Islands: community participation in shaping policy for malaria elimination. Malar J.

[42] WHO (2016). Test procedures for insecticide resistance monitoring in malaria vector mosquitoes.

[43] Report of the eighteenth WHOPES working group meeting. Review of Miranet LN, Pandanet 2.0 LN, Yahe LN, Safenet LN. (Accessed at: http://apps.who.int/iris/bitstream/handle/10665/90976/9789241506304_eng.pdf;jsessionid=FC9BF7661EB8D6824D003AB72EC7B9E7?sequence=1)

[44] Lim SS, Fullman N, Stokes A, Ravishankar N, Masiye F, Murray CJL (2011). Net benefits: a multicountry analysis of observational data examining associations between insecticide-treated mosquito nets and health outcomes. PLoS Med.

